# The effect of COVID-19 lockdown on Iraqi wrestlers

**DOI:** 10.25122/jml-2021-0356

**Published:** 2022-08

**Authors:** Zeyad Tareq Abdulrazzaq

**Affiliations:** 1 Department of Dentistry, Al-Mustafa University College, Baghdad, Iraq

**Keywords:** wrestlers, training, COVID-19, lockdown

## Abstract

The lockdown caused by the COVID-19 pandemic has significantly impacted normal life. At the beginning of March 2020, few cases of infected individuals were recorded, but then the number increased with time, and millions of Iraqi people were forced to stay home, losing many of their daily habits. Banning sports teams from gatherings was a global and local problem facing sports, affecting weight, dietary habits, and other life aspects. This observational retrospective study aimed to determine the changes in athletes' lifestyles like training hours, sleeping hours, weight, and other daily habits. The data were recorded 8 months before the lockdown by their coaches and the medical staff of wrestling teams and after the lockdown by surveys and questionnaires directly from the athletes for this study. The results confirmed that Iraqi wrestlers were still committed to not smoking nor drinking alcohol as before the lockdown, but training hours were down to one hour compared to 3 hours daily before the lockdown. All parts of society were affected by the Covid-19 pandemic, and athletes were on top of the social pyramid to manage the problems that resulted from the quarantine and social distancing. Athletes, coaches, medical sports staff, nutritionists, and captains should carry the responsibility to return life to its main road as they should double their efforts to win this battle.

## INTRODUCTION

Historically, wrestling is thought to have developed from hand-to-hand fighting as a sport. Belt wrestling was seen from 3000 B.C. in Babylonia, Egypt, and the Sumerian cultures in the Epic of Gilgamesh, which depicts such wrestling [1]. The lockdown caused by the COVID-19 pandemic had a massive impact on everyday life, starting with the first cases at the beginning of March 2020. Following this, the number of infected cases and deaths increased with time. In Iraq and some European countries such as Italy and Spain, the government imposed the lockdown as a way to prevent COVID-19 spread, by banning all activities that were not considered essential, such as schools and universities, sports activities, shopping malls, and small shops and factories were closed [2, 3]. Using cars was restricted only to police officers, military forces, and health workers, with some rare exceptions. As a result, millions of Iraqi people were forced to stay home, losing many of their daily habits. Lack of confidence in the health system, uncertainties regarding the cure, no vaccine, and conflicting news on social media were all factors that resulted in sleep disturbance, anxiety, and depressive disorders for almost all individuals. Banning sports teams from gathering was a global and localized problem facing sports [4–10]. Stay-at-home orders resulted in behavioral changes and daily timing exercise changes [11], decreasing the outdoor time that led to increased weight gain. Wrestling is an Olympic fighting sport that involves battling-type techniques with closed restrictions regarding an individual's weight. Therefore, we depended on studies done before and after the pandemic lockdown to evaluate the weight changes of Iraqi wrestlers [12]. The current study aimed to evaluate the changes in wrestlers' lives during the lockdown by comparing their training hours, sleeping hours, weights, and other daily habits data from before and after lockdown.

## Material and Methods

Data was collected through surveys and questionnaires directly obtained for this study. Local healthy athletes with no heart diseases or other chronic disorders were included in this study, aged 25±6 years. Data collection before the quarantine was performed by the medical staff of each team. Data were collected each month to record the progress of the athletes from the national wrestling team of Bagdad. 60 athletes from different weight categories were included in this study. Data collection after 8 months of lockdown was done using questionnaires and surveys. The categories assessed included: weight, smoking, alcohol, narcotics, training hours, night sleeping hours, morning sleeping hours, and food intake (before and after lockdown).

The weights of all athletes were observed regularly and closely by electronic weight scale before the COVID-19 lockdown, and the diets were under the management of specialists. The weights were measured after 8 months of lockdown by the same device. Data on smoking, alcohol, narcotics, training hours, night sleeping hours, morning sleeping hours, and food were collected through self-administered questionnaires and surveys.

## Results

**Figure 1:**
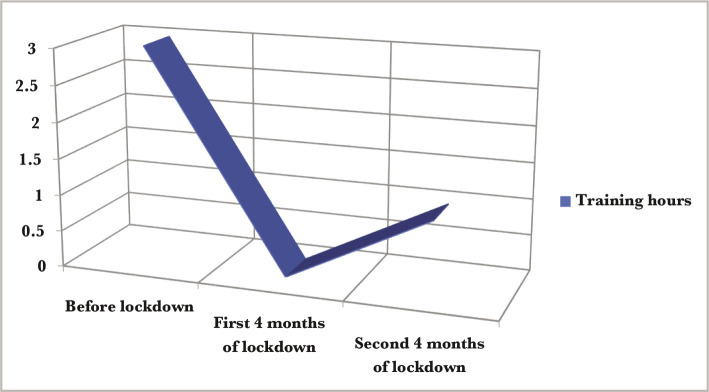
Training during the lockdown period.

**Figure 2:**
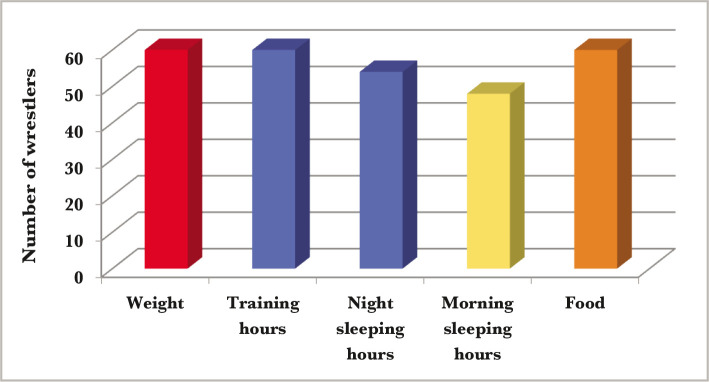
Comparison between the survey questions that the athletes were asked.

**Figure 3:**
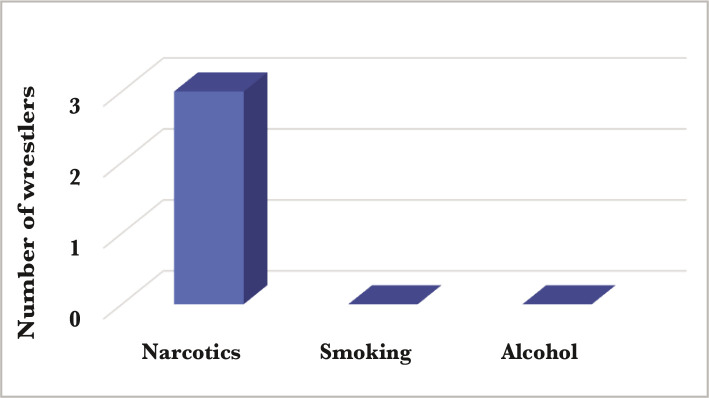
Social habits data after lockdown, data on narcotics, smoking, and alcohol consumption (non-significant).

The results gathered from the surveys (Table 1) showed an increase in the weights of the wrestlers included in the study sample, with an average increase of +5.6 kg over the 8 months of the lockdown. None of the wrestlers started smoking or consumed any alcohol during that period. Only some individuals took narcotics as it helped them fall asleep. For their morning and night sleeping hours, the changes were less night sleep and more morning sleep for 90% and 80% of the wrestlers, respectively. Training hours and fast food decreased after quarantine time (Figures 1–3).

**Table 1:** Rate of change before and after quarantine.

Questioner	Number of wrestlers	Rate of Change after lockdown
**Weight**	100% (60)	+5.6 kg increment
**Smoking**	None (zero)	
**Alcohol**	None (zero)	
**Narcotics**	5% (3)	
**Training hours**	100% (60)	Decreased to 1 hrs.
**Night sleeping hours**	90% (54)	Less
**Morning sleeping hours**	80% (48)	More
**Food**	100% (60)	More unhealthy

## Discussion

During the lockdown, many people were forced to work at home, participate in distance learning, restrict meeting others and isolate as much as possible [13]. Navigation was allowed only for medical emergencies and important work, limited physical activities, and grocery shopping [13]. Both the individual's psychological status and lifestyle behaviors were affected by these critical self-isolation processes and negatively affected people's lives [14–16]. The daily schedule changes and leaving school or college are related to mood changes and could lead to disturbance in eating [17–20]. The results showed a dramatic change in the lifestyle, dietary habits, and overall health of most athletes, reflecting the big impact of the lockdown on our lives. So, we need full cooperation between governmental, civil, and specialized institutions to decrease the effects of this challenge and return life to normal. Using social media guided by coaches' teams can positively impact sports teams.

Furthermore, adult eating habits were disrupted during lockdown due to inactivity [21, 22], severe intestinal lethargy, and other consumption problems [23]. Changes during the Covid-19 lockdown, like inactivity and tiredness, were similar to holiday seasons, when people tend to gain more weight [24, 25]. Longitudinal investigations in China showed that the mean weight gain during one year increased roughly by 0.45 kg [26]. Furthermore, their results showed a considerable weight gain, with the addition of 2.2 kg (95% CI 2.2 to 2.3), over a 4-month lockdown period among the Chinese youth [27]. The change in the individuals' activities, daily bodily movements, and food consumption led to irregular metabolic processes in their bodies, leading to an imbalance in storing and burning fat and calories [27, 28]. For Iraqi wrestlers, weight gain was also due to inactivity hours and changing the usual food system for the athletes. These findings were similar to many European observations [29]. Lack of entertainment, extra free time, online social activities, and online learning increased sedentary time for people staying home during quarantine [29]. It was found that psychological well-being was also affected during lockdown due to the absence of active hours, lack of outdoor work, and social activities [29, 30]. To control weight gain, active work and dietary propensities should be taken into account [31].

## Conclusion

Life should go on in a good way. Although the health staff is the first defender in this crisis, all departments and sections in the community should cooperate to restore the life we had. Athletes and sports managers carry great responsibilities due to their controlled lifestyles. Coaches, medical sports staff, nutritionists, and athletes should double their efforts to win this battle, focusing on teamwork and organization.

## Personal thanks

Special thanks for the cooperation of the National Iraqi Wrestlers team coaches, Muaayad Wahan, Jamal Nasser, and captain Arif Jabbar. Furthermore, we want to thank all the doctors of the wrestling team, medical staff, and the wrestlers of the Iraqi national team.
